# Insights Into the Early Gene Regulatory Network Controlling Neural Crest and Placode Fate Choices at the Neural Border

**DOI:** 10.3389/fphys.2020.608812

**Published:** 2020-11-26

**Authors:** Subham Seal, Anne H. Monsoro-Burq

**Affiliations:** ^1^Université Paris-Saclay, CNRS UMR 3347, INSERM U1021, Orsay, France; ^2^Institut Curie Research Division, PSL Research University, Orsay Cedex, France; ^3^Institut Universitaire de France, Paris, France

**Keywords:** neural border, neural crest, placodes, signaling, gene-regulatory-network, ectoderm patterning, fate decision

## Abstract

The neural crest (NC) cells and cranial placodes are two ectoderm-derived innovations in vertebrates that led to the acquisition of a complex head structure required for a predatory lifestyle. They both originate from the neural border (NB), a portion of the ectoderm located between the neural plate (NP), and the lateral non-neural ectoderm. The NC gives rise to a vast array of tissues and cell types such as peripheral neurons and glial cells, melanocytes, secretory cells, and cranial skeletal and connective cells. Together with cells derived from the cranial placodes, which contribute to sensory organs in the head, the NC also forms the cranial sensory ganglia. Multiple *in vivo* studies in different model systems have uncovered the signaling pathways and genetic factors that govern the positioning, development, and differentiation of these tissues. In this literature review, we give an overview of NC and placode development, focusing on the early gene regulatory network that controls the formation of the NB during early embryonic stages, and later dictates the choice between the NC and placode progenitor fates.

## Introduction

**Figure 1:**
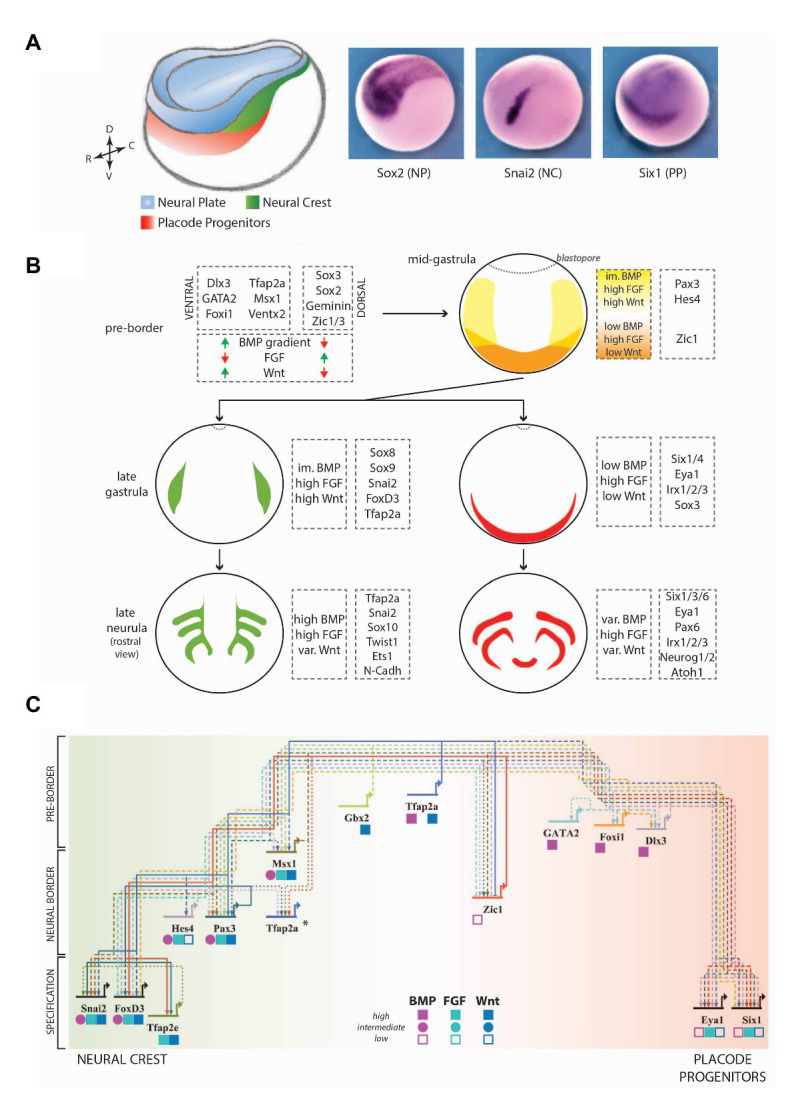
A simplified view of the vertebrate gene regulatory network (GRN) controlling neural crest (NC) and placode induction. **(A)** Model of a *Xenopus* embryo at the mid-neurula stage, depicting the relative positions of the neural plate (NP, blue), the NC (green), and the placode progenitors (PP, red). These tissues express specific transcription factors (TFs), such as Sox2, Snai2, and Six1 respectively. DV, dorsoventral axis; RC, rostrocaudal axis. **(B)** The combined effects of signaling pathways and TFs lead to the development of different tissues in a temporally and spatially regulated manner. Here, the major genes involved at each stage have been indicated, along with the signaling levels of major secreted pathways (BMP, FGF, and WNT). Signaling pathways and genes have been selected according to their conserved functions in various vertebrate animal models and to the availability of detailed studies about their regulation and function in ectoderm patterning. At the mid-gastrula stage (pre-border stage), orange labels the anterior neural border (NB), and yellow depicts the posterior NB. At later stages, green and red depict the NC and the pre-placodal ectoderm respectively. im., intermediate; var., variable. **(C)** A synthetic view of the NB-development GRN in *Xenopus laevis*. Genes have been arranged from top to bottom according to the first stage during which their function is required. Genes positioned towards the left of the map favor the NC fate (green) while genes positioned towards the right of the map favor the PP fate (red). Gene-specific requirements of different signaling pathway activity have been depicted by shapes under the respective gene names (low, intermediate, and high). *Tfap2a has reiterated functions during the different stages, for which it interacts with different binding partners (de Croze et al., 2011; Rothstein and Simoes-Costa, 2020). Solid lines depict direct interactions, dashed lines depict epistasis interactions (either indirect or not proven to be direct) and dotted lines depict a feedback regulation. Arrows depict activation and bars depict repression. The GRN map has been constructed using the BioTapestry software (Longabaugh et al., 2005). Data from other model systems have not been included for the sake of simplicity, but the selected genes broadly display conserved functions in frog and chick. (For more detailed views of placode and NC GRNs, refer to Simoes-Costa and Bronner, 2015; Maharana and Schlosser, 2018; Prasad et al., 2019; Rogers and Nie, 2019; Thiery et al., 2020).

The “New Head” hypothesis (Gans and Northcutt, 1983; Northcutt, 2005) suggests that the presence of a complex head is a significant evolutionary difference between vertebrates and other chordates. During evolution, the vertebrate head has appeared concomitantly with two unique tissues, which are not present (or present in rudimentary form) in earlier-derived organisms: the neural crest (NC) and the sensory placodes. These tissues are formed at the border of the neural fold on the dorsal side of the embryo: placode progenitors (PP) are present rostrally and NC precursors are located more posteriorly (Figure 1A). The NC cells are morphologically distinguishable at the late neurulation stage when they delaminate and migrate away from the edge of the neuroectoderm, towards the final locations where they differentiate (Shellard and Mayor, 2019; Alkobtawi and Monsoro-Burq, 2020; Thiery et al., 2020). In parallel, during neurulation, the pan-placodal ectoderm is subdivided into thickened epithelial areas defining each placode, which contribute to cranial sensory structures (Schlosser, 2008, 2010; Pieper et al., 2011; Grocott el al., 2012; Streit, 2018; Buzzi et al., 2019). Lineage tracing studies have detailed the respective contributions of the NC and the placodes (Noden, 1975; Keller, 1976; Le Douarin, 1980; D’Amico-Martel and Noden, 1983; Couly and Le Douarin, 1985, 1987; Eagleson and Harris, 1990; Garcia-Martinez and Schoenwolf, 1993; Eagleson et al., 1995; Kozlowski et al., 1997; Streit, 2002; Bhattacharya et al., 2004; Xu et al., 2008). Genetic screens conducted in multiple vertebrate species, in particular frog and chick embryos, have identified transcription factors (TFs) which uniquely demarcate NC and PP (Nieto et al., 1994; Ohto et al., 1999; LaBonne and Bronner-Fraser, 2000; Gamill and Bronner-Fraser, 2002; Plouhinec et al., 2014, 2017; Riddiford and Schlosser, 2016; Roellig et al., 2017). NC and PP originate from a common ectodermal domain, located between the dorsal neural plate (NP; future brain and spinal cord) and the ventral non-neural ectoderm (future epidermis), named the “neural border” (NB, also called “neural plate border” elsewhere; Meulemans and Bronner-Fraser, 2004; Groves and LaBonne, 2014; Pla and Monsoro-Burq, 2018; Thiery et al., 2020). At gastrula stages, *pax3/7* genes (*pax3* paralog in *Xenopus* species, *pax7* paralog in chick, and *pax3/7* ancestor gene in lamprey) mark the lateral and posterior NB, but not it’s rostral most portion, while *zic1* marks the anterior NB (Figure 1; Table 1). The formation, positioning, and henceforth specification of the NB into NC and PP are regulated by the coordinated activity of multiple signaling pathways (e.g., FGF, BMP, and WNT pathways) and specific TFs (e.g., *tfap2a/b/c*, *pax3/7*, *zic1*, and *hes4*; Figure 1B). At neurula stages, NC and PP are marked by unique gene sets (e.g., *snai2/foxD3* and *six1/eya1* respectively, Table 1).

**Table 1:** Important references.

References		
	*Xenopus*	Chick
**A. Gene**		
Dlx3/5	Feledy et al., 1999; Luo et al., 2001; Pieper et al., 2012	Pera et al., 1999; McLarren et al., 2003; Khudyakov and Bronner-Fraser, 2009; Linker et al., 2009
Eya1/2	Pieper et al., 2012; Maharana and Schlosser, 2018	McLarren et al., 2003
Foxd3	Monsoro-Burq et al., 2003; Sato et al., 2005; Steventon et al., 2009; Maharana and Schlosser, 2018	Cheung et al., 2005; Khudyakov and Bronner-Fraser, 2009; Simoes-Costa et al., 2012
Foxi1/3	Matsuo-Takasaki et al., 2005; Pieper et al., 2012; Maharana and Schlosser, 2018	Khatri and Groves, 2013
Gata2/3	Pieper et al., 2012; Maharana and Schlosser, 2018	Sheng and Stern, 1999
Gbx2	Li et al., 2009; Steventon and Mayor, 2012	Steventon and Mayor, 2012
Hes4 (Hairy2b)	Nichane et al., 2008a,b; de Croze et al., 2011; Maharana and Schlosser, 2018	
Msx1	Suzuki et al., 1997; Tribulo et al., 2003; Monsoro-Burq et al., 2005	Streit and Stern, 1999; Khudyakov and Bronner-Fraser, 2009; Linker et al., 2009
Pax3/7	Monsoro-Burq et al., 2005; Sato et al., 2005; Hong and St-Jeannet, 2007; de Croze et al., 2011; Milet et al., 2013; Plouhinec et al., 2014; Maharana and Schlosser, 2018	Basch et al., 2006; Otto et al., 2006; Khudyakov and Bronner-Fraser, 2009; Linker et al., 2009; Stuhlmiller and Garcia-Castro, 2012; Vadasz et al., 2013; Simoes-Costa and Bronner, 2015
Six1	Pandur and Moody, 2000; Brugmann et al., 2004; Ahrens and Schlosser, 2005; Pieper et al., 2012; Maharana and Schlosser, 2018	McLarren et al., 2003; Christophorou et al., 2009
Snai2	Mancilla and Mayor, 1996; Monsoro-Burq et al., 2003, 2005; Steventon et al., 2009	Nieto et al., 1994; del Barrio and Nieto, 2002; Khudyakov and Bronner-Fraser, 2009
Tfap2a	Luo et al., 2002, 2003; de Croze et al., 2011; Maharana and Schlosser, 2018	Khudyakov and Bronner-Fraser, 2009; Rothstein and Simoes-Costa, 2020
Tfap2e	Hong et al., 2014	
Zic1	Mizuseki et al., 1998; Monsoro-Burq et al., 2005; Sato et al., 2005; Hong and St-Jeannet, 2007; Marchal et al., 2009; Milet et al., 2013; Plouhinec et al., 2014; Maharana and Schlosser, 2018	Khudyakov and Bronner-Fraser, 2009; Simoes-Costa and Bronner, 2015
**B. Transcriptome analysis**		
	Plouhinec et al., 2014; Riddiford and Schlosser, 2016; Plouhinec et al., 2017; Maharana and Schlosser, 2018	Khudyakov and Bronner-Fraser, 2009; Simoes-Costa et al., 2014; Simoes-Costa and Bronner, 2016; Hintze et al., 2017; Morrison et al., 2017; Roellig et al., 2017; Trevers et al., 2018

In this mini review article, we have gathered as many references as possible and apologize to the authors whose work could not be quoted. We add here a list of additional references for each of the genes described in the text and point to several relevant large-scale transcriptome screening. Studies using frog as a model are indicated in blue, studies using chick embryos in black; A: references describing NC and PP markers; and B: references of transcriptome analysis of NC and PP progenitors.

Principally, the cephalic NC and the placodes form the head sense organs and peripheral nervous system. The cranial NC forms neurons, glial cells, melanocytes, secretory cells, osteocytes, and chondrocytes (Dupin et al., 2018; Etchevers et al., 2019; Alkobtawi and Monsoro-Burq, 2020). The pan-placodal ectoderm develops into non-neurogenic placodes (e.g., adenohypophysis, lens), and neurogenic placodes (epibranchial, otic, paratympanic, trigeminal, and olfactory). In addition, aquatic anamniote vertebrates possess lateral line placodes, which generate a lateral line system comprised of mechanosensory organs in the head and the trunk (Piotrowski and Baker, 2014; Schlosser, 2014; Singh and Groves, 2016; Buzzi et al., 2019). Additionally, by a coordinated migration and morphogenesis, NC, and placode cells form the cranial sensory ganglia (D’Amico-Martel and Noden, 1983; Forni et al., 2011). In humans, defective NC development leads to neurocristopathies, which represent one-third of all developmental diseases, such as cleft palate, Waardenburg syndrome, and Hirschsprung’s disease (Vega-Lopez et al., 2018). Similarly, defects in placode development lead to diseases such as BOR/BO syndrome (Kochhar et al., 2007). In order to understand the development of these tissues and uncover the molecular basis of human pathologies, functional studies have been conducted using various vertebrate animal models. In this brief literature review, we focus on the regulation of the early stages of NB development, followed by its specification into NC and PP. We particularly emphasize the common and specific pathways and the gene regulatory network (GRN) controlling the balanced emergence of both cell types around the NP.

## Neural Crest Development, an Overview

The neural crest is an exclusive feature of vertebrates, acquired about 500 million years ago during evolution (Sauka-Spengler et al., 2007). Since NC generates tissues typical of both ectodermal (ganglia) and mesodermal (mesenchyme, bone) origin, it has been referred to as the fourth embryonic germ layer (Hall, 2018). The NC develops from the NB positioned adjacent to the NP along the rostrocaudal axis during gastrulation and neurulation. Classically, the NC is subdivided into cranial and trunk areas, followed by further anatomical subdivisions (Alkobtawi and Monsoro-Burq, 2020). At the end of neurulation, upon neural tube closure, the NC cells start to migrate in multiple streams, delineating the main craniofacial domains and along the somites in the trunk (Theveneau and Mayor, 2012; Szabo and Mayor, 2018; Rocha et al., 2020). Upon reaching their target tissues, poorly understood genetic programs and interactions with the environment dictate NC differentiation into multiple cell types (Bronner and Le Douarin, 2012).

Before migration, NC cells follow a typical epithelial-to-mesenchymal transition (EMT), which involves the activation of specific TFs (EMT-TFs, e.g., Snail1/2, Twist1), a cadherin switch, and the fine-tuned dynamics of multiple cytoskeletal and cell-polarity proteins. This results in the loss of the polarized epithelial phenotype and acquisition of cell motility (Bahm et al., 2017; Morrison et al., 2017; Shellard and Mayor, 2019). In most species, NC migration involves “contact inhibition of locomotion” (CIL), the mechanism allowing cell dispersion *in vitro* and *in vivo*, as well as “co-attraction,” a mechanism maintaining collective migration of cranial NC cells (Carmona-Fontaine et al., 2008; Wynn et al., 2013; Richardson et al., 2016; Li et al., 2019). In addition, cranial NC cells interact with placodal cells, some of which also delaminate. This helps orient the direction of migration of both cell types (Freter et al., 2013; Theveneau et al., 2013; Colombi et al., 2020). The cellular mechanisms of NC migration have been extensively reviewed elsewhere (Mayor and Theveneau, 2013; Shellard and Mayor, 2019; Alkobtawi and Monsoro-Burq, 2020; Giniunaite et al., 2020; Piacentino et al., 2020; Thiery et al., 2020).

Recent works have focused on premigratory NC induction and specification, starting at late gastrulation/NP stages, as denoted by the expression of early NC specifier genes (e.g., *snai2*, *foxd3*, *tfap2e*, *sox8*, and *sox9*). These earlier NC-specifiers in turn induce later NC specifiers such as *sox10*, *ets1*, and *twist1* during the second half of neurulation, when neural folds elevate and fuse dorsally (Alkobtawi and Monsoro-Burq, 2020). The NC specifiers collectively maintain their own expression by positive feedback stimulations (Lander et al., 2013).

## Placode Development, an Overview

Placodes, the second key vertebrate innovation leading to the formation of specialized head structures, develop from the dorsal-rostral pan-placodal domain which also derives from the NB (Figure 1A). Post neurulation, some placodes undergo epithelial folding. Other placode cells are primed for neurogenesis and delaminate from the epithelium (Lassiter et al., 2014). However, unlike NC migration, placode migration does not seem to involve EMT: EMT markers are absent, and cells do not exhibit a mesenchymal morphology and migrate as neuronal cells through a breach in the basal lamina (Graham et al., 2007). During migration, placode cells interact with specific subpopulations of NC cells to form sensory ganglia.

The Six and Eya family of TFs are the major genes involved in early PP development. At late gastrula stages, Six1/4 and Eya1/2 are induced throughout the PP and are essential for its development (Table 1). These genes are also required at later stages for placode cell-proliferation and neurogenesis (Schlosser et al., 2008). Grown in isolation, PP continues expressing *six1*/*eya2*, but adopts a lens fate “by default,” highlighting that additional regulators control the formation of the other placodes (Bailey et al., 2006). Although, genetic screens have identified a few genes functioning upstream/downstream of the Six/Eya complex, such as Znf462, Homer2, Hes2, Atoh1, the placode GRN remains incompletely understood (Christophorou et al., 2009; Riddiford and Schlosser, 2016; Hintze et al., 2017).

## Regulation of Neural Crest and Placode Fate Specification

Neural crest and PP are specified at late gastrula and neurula stages, while the induction of the NB itself is concomitant to neural induction in dorsal ectoderm, at early gastrula stages (de Crozé et al., 2011). Both these processes are tightly regulated by the activity of signaling pathways and TFs, leading to a strict temporal developmental sequence, resulting in well-defined margins demarcating each tissue.

### Secreted Signaling Pathways Broadly Pattern the Ectoderm

Levels of activity and cross-regulations between BMP, FGF, and WNT signaling pathways are particularly important for the induction of NC and PP, as they initiate spatial subdivisions of the dorsal ectoderm during gastrulation (Wilson and Hemmati-Brivanlou, 1995; Streit and Stern, 1999; Monsoro-Burq et al., 2003; Kudoh et al., 2004; Steventon et al., 2009; Stuhlmiller and Garcia-Castro, 2012; Yardley and Garcia-Castro, 2012; Schille and Schambony, 2017). Activity levels are influenced by the source of ligands and their antagonists. BMP ligands are secreted by the non-neural ectoderm and the ventral mesoderm, while the NP and the organizer produce BMP antagonists (e.g., Noggin, Chordin, Cerberus and Follistatin; Hawley et al., 1995; Wilson and Hemmati-Brivanlou, 1995; Fletcher and Harland, 2008; Patthey et al., 2008; Branney et al., 2009; Linker et al., 2009). This sets up a low-to-high gradient of BMP signaling from the dorsal midline towards the lateral zones. FGF ligands are produced by the paraxial mesoderm, while WNT ligands come from both the paraxial mesoderm and the non-neural ectoderm (Faure et al., 2002; Monsoro-Burq et al., 2003; Steventon et al., 2009). Rostral to the NP, WNT antagonists limit WNT signaling (Pera and De Robertis, 2000; Wilson et al., 2001; Carmona-Fontaine et al., 2007). All these pathways are also modulated temporally as they are required at different levels at multiple stages of neural/NC/PP and epidermis specification. At the early gastrula stage, FGF signaling, along with BMP and WNT antagonists, promotes neural development while high BMP and WNT signaling lead to non-neural ectoderm development (Groves and LaBonne, 2014). Henceforth, FGF/BMP antagonists activate neural factors demarcating the dorsal ectoderm (e.g., *sox2/3*, *otx2*; Streit et al., 2000). BMP activity upregulates the expression of *tfap2a*, *foxi1*, *gata2/3*, and *dlx3/5* in the non-neural ectoderm (Nguyen et al., 1998; Luo et al., 2002; Tribulo et al., 2003; Matsuo-Takasaki et al., 2005; Esterberg and Fritz, 2009; Kwon et al., 2010; de Croze et al., 2011).

Between the neural and non-neural ectoderm, the lateral NB is characterized by high FGF, high WNT, and low to intermediate BMP activity, and uniquely marked by *pax3/7* with an overlapping expression of *tfap2a*, *msx1*, *zic1*, *gbx2*, and *hes4* (Table 1). In contrast, the anterior NB is subjected to high FGF/low BMP/low WNT levels (Figure 1C; Chang and Hemmati-Brivanlou, 1998; Piacentino and Bronner, 2018; Tambalo et al., 2020). The NB is progressively subdivided into NC, PP, dorsal neural tube, and non-neural ectoderm progenitors. Different relative levels of BMP and WNT activity control NC induction and fate maintenance (Steventon et al., 2009; Steventon and Mayor, 2012). It is not yet completely understood how the activity levels of these pathways change dynamically in time and space. One hypothesis is that morphogenesis during neurulation positions the NB close to distinct parts of the mesoderm over time: at mid/late gastrula stages, the dorsal-lateral marginal zone (immature paraxial and intermediate mesoderm precursors) is required for NC induction, while the intermediate mesoderm (pronephros progenitors) maintains NC identity at the early neurula stage. In frog and chick neurula embryos, premigratory NC progenitors exhibit increased BMP activity due to novel signaling modulators (Tribulo et al., 2003; Kwon et al., 2010; Piacentino and Bronner, 2018). Although it remains difficult to compare stages between different species, in zebrafish embryos, a low level of BMP signaling is essential for NC induction while it seems to inhibits PP formation (Nguyen et al., 1998).

Emerging functions of other signaling pathways also contribute to this complex patterning. Retinoic acid signaling contributes to NC induction and migration (Villanueva et al., 2002; Martinez-Morales et al., 2011). Notch signaling is required for *bmp4* and *snail2* expression, regulating NC induction and cell fates at the neural NB (Endo et al., 2002, 2003; Hernandez-Lagunas et al., 2011). AKT signaling is required for premigratory NC induction and maintenance (Sittewelle and Monsoro-Burq, 2018).

### Transcription Factors Control Fate Decisions at the Neural Border

The integration of those multiple signals triggers the activation of specific TFs, which in turn bias NB cells towards a given fate (Figure 1C). Tfap2a and Gbx2, the earliest genes involved in NC induction, both activate *msx1*, *pax3*, and *hes4* (Li et al., 2009; de Croze et al., 2011). Tfap2a is required for both PP (*six1/eya1*) and NC (foxd3) fates (Luo et al., 2003; Kwon et al., 2010; Pieper et al., 2012; Maharana and Schlosser, 2018). In contrast, Gbx2 favors NC fate by inhibiting *six1* expression (Li et al., 2009). Gata2/3 and Foxi TFs (frog *foxi1a* and chick *foxi3*) promote the PP fate by directly activating *six1* expression and also upregulating *dlx3/5* expression (McLarren et al., 2003; Matsuo-Takasaki et al., 2005; Kwon et al., 2010; Sato et al., 2010; Pieper et al., 2012; Khatri et al., 2014; Hintze et al., 2017). Dlx3 (frog) and Dlx5 (chick) are necessary for PP formation through enhancer-mediated activation of *six1* (Sato et al., 2005, 2010). On the other hand, in mouse, chick, and zebrafish, Msx1 inhibits PP fate by repressing *six1* expression, thus promoting NC fate (Zhang et al., 1997; Phillips et al., 2006; Sato et al., 2010). Interestingly, a recent study in *Xenopus* suggests that Msx1 is required for *six1/eya1* expression, as Msx1 depletion slightly decreases *six1* expression, while its overexpression expands *six1/eya1* ectopically (Maharana and Schlosser, 2018). These seemingly contradictory results may be explained by distinct stage-specific requirements for each gene in different experimental settings. Accordingly, it is known that certain genes, like *tfap2a* and *msx1*, are also required for later NC developmental steps (de Croze et al., 2011; Rothstein and Simoes-Costa, 2020). Mechanistically, the Tfap2a protein dimerizes with either Tfap2c or Tfap2b, at NB and NC stage, respectively, to activate different sets of targets (Rothstein and Simoes-Costa, 2020).

The NB marker Pax3 and the more anteriorly localized Zic1 factor are necessary and sufficient for inducing NC and PP in “naive” ectoderm (Monsoro-Burq et al., 2005; Hong and Saint-Jeannet, 2007; Milet et al., 2013; Bae et al., 2014; Plouhinec et al., 2014). *In vivo* and in ectoderm explants, fate choice is controlled by their relative levels: high Pax3 promotes a hatching gland fate (frog-specific ectoderm cell type), high Zic1 promotes PP fate, while a combination of Pax3 and Zic1 promotes NC fate. Zic1 induces PP fate in a Dlx3-dependent manner while Pax3 strongly represses *six1/eya1* expression (Maharana and Schlosser, 2018). Pax3/Zic1 together lead to the direct expression of the NC specifiers *snai1*, *snai2*, and *foxd3* (Milet et al., 2013; Plouhinec et al., 2014; Simoes-Costa et al., 2014). Consequently *in vivo*, during gastrula NB stages, the PP forms in the Zic1-positive/Pax3-negative anterior NB portion, while NC forms in the region where Pax3 and Zic1 overlap. Interestingly, there is some overlap between *pax3/7*-negative and *six1/eya1*-positive areas, thus leading to an interesting conundrum: how are cells sorted in this overlap region? In chick, a few NB cells continue expressing combinations of fate-specific markers until neurula stages and ultimately get sorted into their final domains (Roellig et al., 2017). Future studies considering the temporal and morphogenetic differences in the neurulation between different species will further address this question.

Several recent transcriptomics screens have uncovered novel regulators of NC/PP fate choice (Table 1). For example, in *Xenopus*, *hes4* (*hairy2b*) and *znf703*, expressed broadly at the NB, are required for NC induction. Hes4 upregulates *foxd3*, maintains NC multipotency, and, through the activity of Notch/Delta signaling triggering Id3, promotes NC differentiation (Nagatomo and Hashimoto, 2007; Nichane et al., 2008a,b; de Croze et al., 2011). Znf703, a target of Pax3 and Zic1, is required for NC specifiers expression (Hong and St-Jeannet, 2017; Janesick et al., 2019). In chick, Axud1, a target of WNT signaling, cooperates with NB specifiers Pax7 and Msx1 for NC induction (Simoes-Costa and Bronner, 2015), while Znf462 and Pdlim4 regulate *foxi3* and *dlx5* respectively, affecting PP development (Hintze et al., 2017). These studies highlight the urgent need for functional studies weaving those numerous novel regulators into the current scaffold of the NB-GRN.

## Discussion

Research in multiple model systems has highlighted essential elements of the GRN governing NB induction and NC/PP fate choice (a frog-specific simplified NB-GRN is shown in Figure 1C). Importantly, the functions of the key regulators are largely conserved across species (Table 1). However major questions remain unanswered. Genetic and transcriptome screens show that the NB-GRN is largely incomplete. Moreover, while complex epistasis relationships begin to be established, most direct regulations await a functional validation. Furthermore, complex feed-back and feed-forward mechanisms between signaling pathways and NB specifiers remain incompletely understood (Litsiou et al., 2005; Garnett et al., 2012). BMP signaling activates Tfap2a, Foxi1, and Gata3, which then regulate each other (McLarren et al., 2003; Ahrens and Schlosser, 2005; Litsiou et al., 2005; Kwon et al., 2010; Pieper et al., 2012; Khatri et al., 2014). Gata2 upregulates both BMP and WNT ligands (Sykes et al., 1998). The NB specifiers Pax3, Zic1, Msx1, Hes4, and Tfap2a regulate each other in a feed-forward loop and require additional WNT signaling (Monsoro-Burq et al., 2005; Sato et al., 2005; Maczkowiak et al., 2010; de Croze et al., 2011; Simoes-Costa and Bronner, 2015). Frog PP specifiers *six1/eya1* affect NB and NC specifiers expression (*pax3*, *foxd3*) as well as NB inducers (*tfap2a*, *msx1*, *dlx3*, *gata2*, *foxi1*; Maharana and Schlosser, 2018). As a whole, these complex cross-talk and feedback regulations stabilize fate choices.

Another debated question is how multipotency, a key characteristic of NC and placodes, is controlled during NB development (Baggiolini et al., 2015). Whether high (NC) or more limited (placodes), the diversity of NC/placode derivatives surpasses other cells’ potential at a similar stage and promotes the formation of the New Head. While the molecular basis of placode multipotency remains unexplored, a first model has proposed that NC progenitors retained blastula-type multipotency (Buitrago-Delgado et al., 2015). However, this model is debated since single-cell transcriptomes have shown that the multipotency gene signature proposed by Buitrago-Delgado et al. was not specific to multipotent cells (Briggs et al., 2018). Rather, functional analysis of the vertebrate-specific genetic innovations Nanog/Oct4 (and their orthologs Ventx/Pou5) before or after gastrulation rather suggests that NC progenitors *de novo* activate pluripotency regulators after NB induction (Scerbo and Monsoro-Burq, 2020). This reinitiates multipotency and promotes the ectomesenchyme fate. From an evolutionary perspective, the cranial NB/NC-GRN requires Ventx/Nanog, Pou5/Oct4 and later NC specifier Ets1 to promote jawed structures formation in gnathostomes (Simoes-Costa and Bronner, 2016; Martik et al., 2019; Soldatov et al., 2019; Scerbo and Monsoro-Burq, 2020). Later on, NC specifiers’ downregulation leads to the loss of pluripotency and the initiation of cell differentiation (Dottori et al., 2001; Sasai et al., 2001; Teng et al., 2008; Betancur et al., 2010; Mundell and Labosky, 2011; Dupin et al., 2018).

Despite their limitations, all these studies shed light on the two alternative models proposed for NB development. The “binary competence” model proposes that early in development, the competence to develop either NC or placodes is restricted to the NB and the non-neural ectoderm, respectively (Schlosser, 2008; Pieper et al., 2011, 2012). The “NB” model proposes, that early on, the multipotent NB generates both NC and PP, the relative positions of which are determined at later stages by distinct specifiers. Recent experiments suggest a combination of both models *in vivo*: at blastula to late-gastrula stages, the multipotent NB shows co-expression of markers of either fate and no spatial segregation of fate-biased cells (NB model), but as development proceeds, the capability to form either NC or PP would restrict to subzones of the border (binary competence; Roellig et al., 2017; Briggs et al., 2018; Maharana and Schlosser, 2018). When single-cell transcriptomics studies will explore these early stages with increased resolution in the near future, it will be interesting to re-evaluate how cell lineage choices are controlled at the NB. Altogether, the recent functional analyses of early ectoderm patterning have shed important novel information, increasing knowledge of the GRN acting to promote NC and/or PP for the benefit of future studies of human pathologies.

## Author Contributions

All authors listed have made a substantial, direct and intellectual contribution to the work, and approved it for publication.

### Conflict of Interest

The authors declare that the research was conducted in the absence of any commercial or financial relationships that could be construed as a potential conflict of interest.

## References

[ref1] Ahrens K. Schlosser G. (2005). Tissues and signals involved in the induction of placodal Six1 expression in *Xenopus laevis*. Dev. Biol. 288, 40–59. 10.1016/j.ydbio.2005.07.022, PMID: 16271713

[ref2] Alkobtawi M. Monsoro-Burq A. H. (2020). “Chapter 1: The neural crest, a vertebrate invention” in Evolving neural crest cells. 1st Edn. eds. Eames B. F. Medeiros D. M. Adameyko I. (Boca Raton: Imprint CRC Press), 5–66.

[ref3] Bae C. J. Park B. Y. Lee Y. H. Tobias J. W. Hong C. S. Saint-Jeannet J. P. (2014). Identification of Pax3 and Zic1 targets in the developing neural crest. Dev. Biol. 386, 473–483. 10.1016/j.ydbio.2013.12.011, PMID: 24360908 PMC3933997

[ref4] Baggiolini A. Varum S. Mateos J. M. Bettosini D. John N. Bonalli M.. (2015). Premigratory and migratory neural crest cells are multipotent in vivo. Cell Stem Cell 16, 314–322. 10.1016/j.stem.2015.02.017, PMID: 25748934

[ref5] Bahm I. Barriga E. H. Frolov A. Theveneau E. Frankel P. Mayor R. (2017). PDGF controls contact inhibition of locomotion by regulating N-cadherin during neural crest migration. Development 144, 2456–2468. 10.1242/dev.147926, PMID: 28526750 PMC5536867

[ref6] Bailey A. P. Bhattacharyya S. Bronner-Fraser M. Streit A. (2006). Lens specification is the ground state of all sensory placodes, from which FGF promotes olfactory identity. Dev. Cell 11, 505–517. 10.1016/j.devcel.2006.08.009, PMID: 17011490

[ref154] Basch M. L. Bronner-Fraser M. García-Castro M. I. (2006). Specification of the neural crest occurs during gastrulation and requires Pax7. Nature 441, 218–222. 10.1038/nature04684, PMID: 16688176

[ref7] Betancur P. Bronner-Fraser M. Sauka-Spengler T. (2010). Assembling neural crest regulatory circuits into a gene regulatory network. Annu. Rev. Cell Dev. Biol. 26, 581–603. 10.1146/annurev.cellbio.042308.113245, PMID: 19575671 PMC4040144

[ref8] Bhattacharyya S. Bailey A. P. Bronner-Fraser M. Streit A. (2004). Segregation of lens and olfactory precursors from a common territory: cell sorting and reciprocity of Dlx5 and Pax6 expression. Dev. Biol. 271, 403–414. 10.1016/j.ydbio.2004.04.010, PMID: 15223343

[ref9] Branney P. A. Faas L. Steane S. E. Pownall M. E. Isaacs H. V. (2009). Characterisation of the fibroblast growth factor dependent transcriptome in early development. PLoS One 4:e4951. 10.1371/journal.pone.0004951, PMID: 19333377 PMC2659300

[ref10] Briggs J. A. Weinreb C. Wagner D. E. Megason S. Peshkin L. Kirschner M. W.. (2018). The dynamics of gene expression in vertebrate embryogenesis at single-cell resolution. Science 360:eaar5780. 10.1126/science.aar5780, PMID: 29700227 PMC6038144

[ref11] Bronner M. E. LeDouarin N. M. (2012). Development and evolution of the neural crest: an overview. Dev. Biol. 366, 2–9. 10.1016/j.ydbio.2011.12.042, PMID: 22230617 PMC3351559

[ref12] Brugmann S. A. Pandur P. D. Kenyon K. L. Pignoni F. Moody S. A. (2004). Six1 promotes a placodal fate within the lateral neurogenic ectoderm by functioning as both a transcriptional activator and repressor. Development 131, 5871–5881. 10.1242/dev.01516, PMID: 15525662

[ref13] Buitrago-Delgado E. Nordin K. Rao A. Geary L. LaBonne C. (2015). Shared regulatory programs suggest retention of blastula-stage potential in neural crest cells. Science 348, 1332–1335. 10.1126/science.aaa3655, PMID: 25931449 PMC4652794

[ref14] Buzzi A. L. Hintze M. S. Streit A. (2019). “Development of neurogenic placodes in vertebrates” in eLS. ed. Wiley J. (Chichester, UK: John Wiley * Sons, Ltd.), 1–14.

[ref15] Carmona-Fontaine C. Acuña G. Ellwanger K. Niehrs C. Mayor R. (2007). Neural crests are actively precluded from the anterior neural fold by a novel inhibitory mechanism dependent on Dickkopf1 secreted by the prechordal mesoderm. Dev. Biol. 309, 208–221. 10.1016/j.ydbio.2007.07.006, PMID: 17669393

[ref16] Carmona-Fontaine C. Matthews H. K. Kuriyama S. Moreno M. Dunn G. A. Parsons M.. (2008). Contact inhibition of locomotion in vivo controls neural crest directional migration. Nature 456, 957–961. 10.1038/nature07441, PMID: 19078960 PMC2635562

[ref17] Chang C. Hemmati-Brivanlou A. (1998). Neural crest induction by Xwnt7B in *Xenopus*. Dev. Biol. 194, 129–134. 10.1006/dbio.1997.8820, PMID: 9473337

[ref18] Cheung M. Chaboissier M. C. Mynett A. Hirst E. Schedl A. Briscoe J. (2005). The transcriptional control of trunk neural crest induction, survival, and delamination. Dev. Cell 8, 179–192. 10.1016/j.devcel.2004.12.010, PMID: 15691760

[ref19] Christophorou N. A. D. Bailey A. P. Hanson S. Streit A. (2009). Activation of Six1 target genes is required for sensory placode formation. Dev. Biol. 336, 327–336. 10.1016/j.ydbio.2009.09.025, PMID: 19781543

[ref20] Colombi A. Scianna M. Painter K. J. Preziosi L. (2020). Modelling chase-and-run migration in heterogeneous populations. J. Math. Biol. 80, 423–456. 10.1007/s00285-019-01421-9, PMID: 31468116 PMC7012813

[ref21] Couly G. F. Le Douarin N. M. (1985). Mapping of the early neural primordium in quail-chick chimeras. I. Developmental relationships between placodes, facial ectoderm, and prosencephalon. Dev. Biol. 110, 422–439. 10.1016/0012-1606(85)90101-0, PMID: 4018406

[ref22] Couly G. F. Le Douarin N. M. (1987). Mapping of the early neural primordium in quail-chick chimeras. II. The prosencephalic neural plate and neural folds: implications for the genesis of cephalic human congenital abnormalities. Dev. Biol. 120, 198–214. 10.1016/0012-1606(87)90118-7, PMID: 3817289

[ref23] D’amico-Martel A. Noden D. M. (1983). Contributions of placodal and neural crest cells to avian cranial peripheral ganglia. Am. J. Anat. 166, 445–468. 10.1002/aja.1001660406, PMID: 6858941

[ref24] De Crozé N. Maczkowiak F. Monsoro-Burq A. H. (2011). Reiterative AP2a activity controls sequential steps in the neural crest gene regulatory network. Proc. Natl. Acad. Sci. U. S. A. 108, 155–160. 10.1073/pnas.1010740107, PMID: 21169220 PMC3017139

[ref25] del Barrio M. G. Nieto M. A. (2002). Overexpression of Snail family members highlights their ability to promote chick neural crest formation. Development 129, 1583–1593. PMID: 11923196 10.1242/dev.129.7.1583

[ref26] Dottori M. Gross M. K. Labosky P. Goulding M. (2001). The winged-helix transcription factor Foxd3 suppresses interneuron differentiation and promotes neural crest cell fate. Development 128, 4127–4138. PMID: 11684651 10.1242/dev.128.21.4127

[ref27] Dupin E. Calloni G. W. Coelho-Aguiar J. M. Le Douarin N. M. (2018). The issue of the multipotency of the neural crest cells. Dev. Biol. 444, S47–S59. 10.1016/j.ydbio.2018.03.024, PMID: 29614271

[ref28] Eagleson G. Ferreiro B. Harris W. A. (1995). Fate of the anterior neural ridge and the morphogenesis of the *Xenopus* forebrain. J. Neurobiol. 28, 146–158. 10.1002/neu.480280203, PMID: 8537821

[ref29] Eagleson G. W. Harris W. A. (1990). Mapping of the presumptive brain regions in the neural plate of *Xenopus laevis*. J. Neurobiol. 21, 427–440. 10.1002/neu.480210305, PMID: 2351962

[ref30] Endo Y. Osumi N. Wakamatsu Y. (2002). Bimodal functions of Notch-mediated signaling are involved in neural crest formation during avian ectoderm development. Development 129, 863–873. PMID: 11861470 10.1242/dev.129.4.863

[ref31] Endo Y. Osumi N. Wakamatsu Y. (2003). Deltex/Dtx mediates NOTCH signaling in regulation of Bmp4 expression in cranial neural crest formation during avian development. Develop. Growth Differ. 45, 241–248. 10.1046/j.1524-4725.2003.693.x, PMID: 12828685

[ref32] Esterberg R. Fritz A. (2009). dlx3b/4b are required for the formation of the preplacodal region and otic placode through local modulation of BMP activity. Dev. Biol. 325, 189–199. 10.1016/j.ydbio.2008.10.017, PMID: 19007769 PMC2674874

[ref33] Etchevers H. C. Dupin E. Le Douarin N. M. (2019). The diverse neural crest: from embryology to human pathology. Dev. 146:dev169821. 10.1242/dev.169821, PMID: 30858200

[ref34] Faure S. De Santa Barbara P. Roberts D. J. Whitman M. (2002). Endogenous patterns of BMP signaling during early chick development. Dev. Biol. 244, 44–65. 10.1006/dbio.2002.0579, PMID: 11900458

[ref35] Feledy J. A. Beanan M. J. Sandoval J. J. Goodrich J. S. Lim J. H. Matsuo-Takasaki M.. (1999). Inhibitory patterning of the anterior neural plate in *Xenopus* by homeodomain factors D1x3 and Msx1. Dev. Biol. 212, 455–464. 10.1006/dbio.1999.9374, PMID: 10433834

[ref36] Fletcher R. B. Harland R. M. (2008). The role of FGF signaling in the establishment and maintenance of mesodermal gene expression in *Xenopus*. Dev. Dyn. 237, 1243–1254. 10.1002/dvdy.21517, PMID: 18386826 PMC3000043

[ref37] Forni P. E. Taylor-Burds C. Melvin V. S. Williams T. Wray S. (2011). Neural crest and ectodermal cells intermix in the nasal placode to give rise to GnRH-1 neurons, sensory neurons, and olfactory ensheathing cells. J. Neurosci. 31, 6915–6927. 10.1523/JNEUROSCI.6087-10.2011, PMID: 21543621 PMC3101109

[ref38] Freter S. Fleenor S. J. Freter R. Liu K. J. Begbie J. (2013). Cranial neural crest cells form corridors prefiguring sensory neuroblast migration. Development 140, 3595–3600. 10.1242/dev.091033, PMID: 23942515 PMC3742142

[ref39] Gammill L. S. Bronner-Fraser M. (2002). Genomic analysis of neural crest induction. Development 129, 5731–5741. 10.1242/dev.00175, PMID: 12421712

[ref40] Gans C. Northcutt R. G. (1983). Neural crest and the origin of vertebrates: a new head. Science 220, 268–274. 10.1126/science.220.4594.268, PMID: 17732898

[ref155] Garcia-Martinez V. Schoenwolf G. C. (1993). Primitive-streak origin of the cardiovascular system in avian embryos. Dev. Biol. 159, 706–719. 10.1006/dbio.1993.1276, PMID: 8405690

[ref41] Garnett A. T. Square T. A. Medeiros D. M. (2012). BMP, wnt and FGF signals are integrated through evolutionarily conserved enhancers to achieve robust expression of Pax3 and Zic genes at the zebrafish neural plate border. Development 139, 4220–4231. 10.1242/dev.081497, PMID: 23034628 PMC4074300

[ref42] Giniūnaitė R. McLennan R. McKinney M. C. Baker R. E. Kulesa P. M. Maini P. K. (2020). An interdisciplinary approach to investigate collective cell migration in neural crest. Dev. Dyn. 249, 270–280. 10.1002/dvdy.124, PMID: 31622517

[ref43] Graham A. Blentic A. Duque S. Begbie J. (2007). Delamination of cells from neurogenic placodes does not involve an epithelial-to-mesenchymal transition. Development 134, 4141–4145. 10.1242/dev.02886, PMID: 17959723

[ref44] Grocott T. Tambalo M. Streit A. (2012). The peripheral sensory nervous system in the vertebrate head: a gene regulatory perspective. Dev. Biol. 370, 3–23. 10.1016/j.ydbio.2012.06.028, PMID: 22790010

[ref45] Groves A. K. LaBonne C. (2014). Setting appropriate boundaries: fate, patterning and competence at the neural plate border. Dev. Biol. 389, 2–12. 10.1016/j.ydbio.2013.11.027, PMID: 24321819 PMC3972267

[ref46] Hall B. K. (2018). Germ layers, the neural crest and emergent organization in development and evolution. Genesis 56:e23103. 10.1002/dvg.23103, PMID: 29637683

[ref47] Hawley S. H. B. Wünnenberg-Stapleton K. Hashimoto C. Laurent M. N. Watabe T. Blumberg B. W.. (1995). Disruption of BMP signals in embryonic *Xenopus* ectoderm leads to direct neural induction. Genes Dev. 9, 2923–2935. 10.1101/gad.9.23.2923, PMID: 7498789

[ref48] Hernandez-Lagunas L. Powell D. R. Law J. Grant K. A. Artinger K. B. (2011). Prdm1a and Olig4 act downstream of NOTCH signaling to regulate cell fate at the neural plate border. Dev. Biol. 356, 496–505. 10.1016/j.ydbio.2011.06.005, PMID: 21689645 PMC3144709

[ref49] Hintze M. Prajapati R. S. Tambalo M. Christophorou N. A. D. Anwar M. Grocott T.. (2017). Cell interactions, signals and transcriptional hierarchy governing placode progenitor induction. Dev. 144, 2810–2823. 10.1242/dev.147942, PMID: 28684624 PMC5560042

[ref50] Hong C. S. Devotta A. Lee Y. H. Park B. Y. Saint-Jeannet J. P. (2014). Transcription factor AP2 epsilon (Tfap2e) regulates neural crest specification in *Xenopus*. Dev. Neurobiol. 74, 894–906. 10.1002/dneu.22173, PMID: 24616412 PMC4107115

[ref51] Hong C. S. Saint-Jeannet J. P. (2007). The activity of Pax3 and Zic1 regulates three distinct cell fates at the neural plate border. Mol. Biol. Cell 18, 2192–2202. 10.1091/mbc.E06-11-1047, PMID: 17409353 PMC1877120

[ref52] Hong C. S. Saint-Jeannet J. P. (2017). Znf703, a novel target of Pax3 and Zic1, regulates hindbrain and neural crest development in *Xenopus*. Genesis 55:e23082. 10.1002/dvg.23082, PMID: 29086464 PMC5734999

[ref53] Janesick A. Tang W. Ampig K. Blumberg B. (2019). Znf703 is a novel RA target in the neural plate border. Sci. Rep. 9:8275. 10.1038/s41598-019-44722-1, PMID: 31164691 PMC6547707

[ref54] Keller R. E. (1976). Vital dye mapping of the gastrula and neurula of Xenopus laevis. II. Prospective areas and morphogenetic movements of the deep layer. Dev. Biol. 51, 118–137. 10.1016/0012-1606(76)90127-5, PMID: 950072

[ref55] Khatri S. B. Edlund R. K. Groves A. K. (2014). Foxi3 is necessary for the induction of the chick otic placode in response to FGF signaling. Dev. Biol. 391, 158–169. 10.1016/j.ydbio.2014.04.014, PMID: 24780628 PMC4070591

[ref156] Khatri S. B. Groves A. K. (2013). Expression of the Foxi2 and Foxi3 transcription factors during development of chicken sensory placodes and pharyngeal arches. Gene Expr. Patterns 13, 38–42. 10.1016/j.gep.2012.10.001 23124078 PMC3562376

[ref56] Khudyakov J. Bronner-Fraser M. (2009). Comprehensive spatiotemporal analysis of early chick neural crest network genes. Dev. Dyn. 238, 716–723. 10.1002/dvdy.21881, PMID: 19235729 PMC2650819

[ref57] Kochhar A. Fischer S. M. Kimberling W. J. Smith R. J. H. (2007). Branchio-oto-renal syndrome. Am. J. Med. Genet 143, 1671–1678. 10.1002/ajmg.a.31561, PMID: 17238186

[ref58] Kozlowski D. J. Murakami T. Ho R. K. Weinberg E. S. (1997). Regional cell movement and tissue patterning in the zebrafish embryo revealed by fate mapping with caged fluorescein. Biochem. Cell Biol. 75, 551–562. 10.1139/o97-090, PMID: 9551179

[ref59] Kudoh T. Concha M. L. Houart C. Dawid I. B. Wilson S. W. (2004). Combinatorial Fgf and Bmp signalling patterns the gastrula ectoderm into prospective neural and epidermal domains. Development 131, 3581–3592. 10.1242/dev.01227, PMID: 15262889 PMC2789263

[ref60] Kwon H. J. Bhat N. Sweet E. M. Cornell R. A. Riley B. B. (2010). Identification of early requirements for preplacodal ectoderm and sensory organ development. PLoS Genet. 6:e1001133. 10.1371/journal.pgen.1001133, PMID: 20885782 PMC2944784

[ref61] Labonne C. Bronner-Fraser M. (2000). Snail-related transcriptional repressors are required in *Xenopus* for both the induction of the neural crest and its subsequent migration. Dev. Biol. 221, 195–205. 10.1006/dbio.2000.9609, PMID: 10772801

[ref62] Lander R. Nasr T. Ochoa S. D. Nordin K. Prasad M. S. Labonne C. (2013). Interactions between Twist and other core epithelial-mesenchymal transition factors are controlled by GSK3-mediated phosphorylation. Nat. Commun. 4:1542. 10.1038/ncomms2543, PMID: 23443570 PMC4198179

[ref63] Lassiter R. N. T. Stark M. R. Zhao T. Zhou C. J. (2014). Signaling mechanisms controlling cranial placode neurogenesis and delamination. Dev. Biol. 389, 39–49. 10.1016/j.ydbio.2013.11.025, PMID: 24315854 PMC3972360

[ref157] Le Douarin N. M. (1980). The ontogeny of the neural crest in avian embryo chimaeras. Nature 286, 663–669. 10.1038/286663a0, PMID: 6106161

[ref64] Li B. Kuriyama S. Moreno M. Mayor R. (2009). The posteriorizing gene Gbx2 is a direct target of WNT signalling and the earliest factor in neural crest induction. Development 136, 3267–3278. 10.1242/dev.036954, PMID: 19736322 PMC2808295

[ref65] Li Y. Vieceli F. M. Gonzalez W. G. Li A. Tang W. Lois C.. (2019). In vivo quantitative imaging provides insights into trunk neural crest migration. Cell Rep. 26, 1489.e3–1500.e3. 10.1016/j.celrep.2019.01.039, PMID: 30726733 PMC6449054

[ref66] Linker C. De Almeida I. Papanayotou C. Stower M. Sabado V. Ghorani E.. (2009). Cell communication with the neural plate is required for induction of neural markers by BMP inhibition: evidence for homeogenetic induction and implications for *Xenopus* animal cap and chick explant assays. Dev. Biol. 327, 478–486. 10.1016/j.ydbio.2008.12.034, PMID: 19162002 PMC2713608

[ref67] Litsiou A. Hanson S. Streit A. (2005). A balance of FGF, BMP and WNT signaling positions the future placode territory in the head. Development 132, 4051–4062. 10.1242/dev.01964, PMID: 16093325

[ref158] Longabaugh W. J. R. Davidson E. H. Bolouri H. (2005). Computational representation of developmental genetic regulatory networks. Dev. Biol. 283, 1–16. 10.1016/j.ydbio.2005.04.023, PMID: 15907831

[ref68] Luo T. Lee Y. H. Saint-Jeannet J. P. Sargent T. D. (2003). Induction of neural crest in *Xenopus* by transcription factor AP2α. Proc. Natl. Acad. Sci. U. S. A. 100, 532–537. 10.1073/pnas.0237226100, PMID: 12511599 PMC141030

[ref69] Luo T. Matsuo-Takasaki M. Sargent T. D. (2001). Distinct roles for distal-less genes dlx3 and dlx5 in regulating ectodermal development in *Xenopus*. Mol. Reprod. Dev. 60, 331–337. 10.1002/mrd.1095, PMID: 11599044

[ref70] Luo T. Matsuo-Takasaki M. Thomas M. L. Weeks D. L. Sargent T. D. (2002). Transcription factor AP-2 is an essential and direct regulator of epidermal development in *Xenopus*. Dev. Biol. 245, 136–144. 10.1006/dbio.2002.0621, PMID: 11969261

[ref71] Maczkowiak F. Matéos S. Wang E. Roche D. Harland R. Monsoro-Burq A. H. (2010). The Pax3 and Pax7 paralogs cooperate in neural and neural crest patterning using distinct molecular mechanisms, in *Xenopus laevis* embryos. Dev. Biol. 340, 381–396. 10.1016/j.ydbio.2010.01.022, PMID: 20116373 PMC3755748

[ref72] Maharana S. K. Schlosser G. (2018). A gene regulatory network underlying the formation of pre-placodal ectoderm in *Xenopus laevis*. BMC Biol. 16:79. 10.1186/s12915-018-0540-5, PMID: 30012125 PMC6048776

[ref73] Mancilla A. Mayor R. (1996). Neural crest formation in *Xenopus laevis*: mechanisms of Xslug induction. Dev. Biol. 177, 580–589. 10.1006/dbio.1996.0187, PMID: 8806833

[ref74] Marchal L. Luxardi G. Thomé V. Kodjabachian L. (2009). BMP inhibition initiates neural induction via FGF signaling and Zic genes. Proc. Natl. Acad. Sci. U. S. A. 106, 17437–17442. 10.1073/pnas.0906352106, PMID: 19805078 PMC2765096

[ref75] Martik M. L. Gandhi S. Uy B. R. Gillis J. A. Green S. A. Simoes-Costa M.. (2019). Evolution of the new head by gradual acquisition of neural crest regulatory circuits. Nature 574, 675–678. 10.1038/s41586-019-1691-4, PMID: 31645763 PMC6858584

[ref76] Martínez-Morales P. L. del Corral R. D. Olivera-Martínez I. Quiroga A. C. Das R. M. Barbas J. A.. (2011). FGF and retinoic acid activity gradients control the timing of neural crest cell emigration in the trunk. J. Cell Biol. 194, 489–503. 10.1083/jcb.201011077, PMID: 21807879 PMC3153641

[ref77] Matsuo-Takasaki M. Matsumura M. Sasai Y. (2005). An essential role of *Xenopus* Foxi1a for ventral specification of the cephalic ectoderm during gastrulation. Development 132, 3885–3894. 10.1242/dev.01959, PMID: 16079156

[ref159] Mayor R. Theveneau E. (2013). The neural crest. Development 140, 2247–2251. 10.1242/dev.091751, PMID: 23674598

[ref78] McLarren K. W. Litsiou A. Streit A. (2003). Dlx5 positions the neural crest and preplacode region at the border of the neural plate. Dev. Biol. 259, 34–47. 10.1016/S0012-1606(03)00177-5, PMID: 12812786

[ref79] Meulemans D. Bronner-Fraser M. (2004). Gene-regulatory interactions in neural crest evolution and development. Dev. Cell 7, 291–299. 10.1016/j.devcel.2004.08.007, PMID: 15363405

[ref80] Milet C. Maczkowiak F. Roche D. D. Monsoro-Burq A. H. (2013). Pax3 and Zic1 drive induction and differentiation of multipotent, migratory, and functional neural crest in *Xenopus* embryos. Proc. Natl. Acad. Sci. 110, 5528–5533. 10.1073/pnas.1219124110, PMID: 23509273 PMC3619367

[ref160] Mizuseki K. Kishi M. Matsui M. Nakanishi S. Sasai Y. (1998). Xenopus Zic-related-1 and Sox-2, two factors induced by chordin, have distinct activities in the initiation of neural induction. Development 125, 579–587. PMID: 9435279 10.1242/dev.125.4.579

[ref81] Monsoro-Burq A. H. Fletcher R. B. Harland R. M. (2003). Neural crest induction by paraxial mesoderm in *Xenopus* embryos requires FGF signals. Development 130, 3111–3124. 10.1242/dev.00531, PMID: 12783784

[ref82] Monsoro-Burq A. H. Wang E. Harland R. (2005). Msx1 and Pax3 cooperate to mediate FGF8 and WNT signals during *Xenopus* neural crest induction. Dev. Cell 8, 167–178. 10.1016/j.devcel.2004.12.017, PMID: 15691759

[ref83] Morrison J. A. McLennan R. Wolfe L. A. Gogol M. M. Meier S. McKinney M. C.. (2017). Single-cell transcriptome analysis of avian neural crest migration reveals signatures of invasion and molecular transitions. eLife 6:e28415. 10.7554/eLife.28415, PMID: 29199959 PMC5728719

[ref84] Mundell N. A. Labosky P. A. (2011). Neural crest stem cell multipotency requires Foxd3 to maintain neural potential and repress mesenchymal fates. J. Cell Sci. 124, 641–652. 10.1242/jcs.086983, PMID: 21228004 PMC3026411

[ref85] Nagatomo K. -I. Hashimoto C. (2007). *Xenopus* hairy2 functions in neural crest formation by maintaining cells in a mitotic and undifferentiated state. Dev. Dyn. 236, 1475–1483. 10.1002/dvdy.21152, PMID: 17436284

[ref86] Nguyen V. H. Schmid B. Trout J. Connors S. A. Ekker M. Mullins M. C. (1998). Ventral and lateral regions of the zebrafish gastrula, including the neural crest progenitors, are established by a Bmp2b/Swirl pathway of genes. Dev. Biol. 199, 93–110. 10.1006/dbio.1998.8927, PMID: 9676195

[ref87] Nichane M. de Crozé N. Ren X. Souopgui J. Monsoro-Burq A. H. Bellefroid E. J. (2008a). Hairy2-Id3 interactions play an essential role in *Xenopus* neural crest progenitor specification. Dev. Biol. 322, 355–367. 10.1016/j.ydbio.2008.08.003, PMID: 18721802

[ref88] Nichane M. Ren X. Souopgui J. Bellefroid E. J. (2008b). Hairy2 functions through both DNA-binding and non DNA-binding mechanisms at the neural plate border in *Xenopus*. Dev. Biol. 322, 368–380. 10.1016/j.ydbio.2008.07.026, PMID: 18710660

[ref89] Nieto M. A. Sargent M. G. Wilkinson D. G. Cooke J. (1994). Control of cell behavior during vertebrate development by Slug, a zinc finger gene. Science 264, 835–839. 10.1126/science.7513443, PMID: 7513443

[ref90] Noden D. M. (1975). An analysis of the migratory behavior of avian cephalic neural crest cells. Dev. Biol. 42, 106–130. 10.1016/0012-1606(75)90318-8, PMID: 1112437

[ref91] Northcutt R. G. (2005). The new head hypothesis revisited. J. Exp. Zool. B Mol. Dev. Evol. 304, 274–297. 10.1002/jez.b.21063, PMID: 16003768

[ref92] Ohto H. Kamada S. Tago K. Tominaga S. -I. Ozaki H. Sato S.. (1999). Cooperation of six and Eya in activation of their target genes through nuclear translocation of Eya. Mol. Cell. Biol. 19, 6815–6824. 10.1128/mcb.19.10.6815, PMID: 10490620 PMC84678

[ref93] Otto A. Schmidt C. Patel K. (2006). Pax3 and Pax7 expression and regulation in the avian embryo. Anat. Embryol. 211, 293–310. 10.1007/s00429-006-0083-3, PMID: 16506066

[ref94] Pandur P. D. Moody S. A. (2000). *Xenopus* Six1 gene is expressed in neurogenic cranial placodes and maintained in the differentiating lateral lines. Mech. Dev. 96, 253–257. 10.1016/S0925-4773(00)00396-8, PMID: 10960794

[ref95] Patthey C. Gunhaga L. Edlund T. (2008). Early development of the central and peripheral nervous systems is coordinated by WNT and BMP signals. PLoS One 3:e1625. 10.1371/journal.pone.0001625, PMID: 18286182 PMC2229838

[ref96] Pera E. Stein S. Kessel M. (1999). Ectodermal patterning in the avian embryo: epidermis versus neural plate. Development 126, 63–73. PMID: 9834186 10.1242/dev.126.1.63

[ref161] Pera E. M. De Robertis E. M. (2000). A direct screen for secreted proteins in *Xenopus* embryos identifies distinct activities for the Wnt antagonists Crescent and Frzb-1. Mech. Dev. 96, 183–195. 10.1016/S0925-4773(00)00394-4 10960783

[ref97] Phillips B. T. Kwon H. J. Melton C. Houghtaling P. Fritz A. Riley B. B. (2006). Zebrafish msxB, msxC and msxE function together to refine the neural-nonneural border and regulate cranial placodes and neural crest development. Dev. Biol. 294, 376–390. 10.1016/j.ydbio.2006.03.001, PMID: 16631154

[ref98] Piacentino M. L. Bronner M. E. (2018). Intracellular attenuation of BMP signaling via CKIP-1/Smurf1 is essential during neural crest induction. PLoS Biol. 16:e2004425. 10.1371/journal.pbio.2004425, PMID: 29949573 PMC6039030

[ref99] Piacentino M. L. Li Y. Bronner M. E. (2020). Epithelial-to-mesenchymal transition and different migration strategies as viewed from the neural crest. Curr. Opin. Cell Biol. 66, 43–50. 10.1016/j.ceb.2020.05.001, PMID: 32531659 PMC7578021

[ref100] Pieper M. Ahrens K. Rink E. Peter A. Schlosser G. (2012). Differential distribution of competence for panplacodal and neural crest induction to non-neural and neural ectoderm. Development 139, 1175–1187. 10.1242/dev.074468, PMID: 22318231

[ref101] Pieper M. Eagleson G. W. Wosniok W. Schlosser G. (2011). Origin and segregation of cranial placodes in *Xenopus laevis*. Dev. Biol. 360, 257–275. 10.1016/j.ydbio.2011.09.024, PMID: 21989028

[ref102] Piotrowski T. Baker C. V. H. (2014). The development of lateral line placodes: taking a broader view. Dev. Biol. 389, 68–81. 10.1016/j.ydbio.2014.02.016, PMID: 24582732

[ref103] Pla P. Monsoro-Burq A. H. (2018). The neural border: induction, specification and maturation of the territory that generates neural crest cells. Dev. Biol. 444, S36–S46. 10.1016/j.ydbio.2018.05.018, PMID: 29852131

[ref104] Plouhinec J. L. Medina-Ruiz S. Borday C. Bernard E. Vert J. P. Eisen M. B.. (2017). A molecular atlas of the developing ectoderm defines neural, neural crest, placode, and nonneural progenitor identity in vertebrates. PLoS Biol. 15:e2004045. 10.1371/journal.pbio.2004045, PMID: 29049289 PMC5663519

[ref105] Plouhinec J. L. Roche D. D. Pegoraro C. Figueiredo A. L. Maczkowiak F. Brunet L. J.. (2014). Pax3 and Zic1 trigger the early neural crest gene regulatory network by the direct activation of multiple key neural crest specifiers. Dev. Biol. 386, 461–472. 10.1016/j.ydbio.2013.12.010, PMID: 24360906 PMC3962137

[ref162] Prasad M. S. Charney R. M. García-Castro M. I. (2019). Specification and formation of the neural crest: perspectives on lineage segregation. Genesis 57:e23276. 10.1002/dvg.23276 30576078 PMC6570420

[ref163] Richardson J. Gauert A. Briones Montecinos L. Fanlo L. Alhashem Z. M. Assar R.. (2016). Leader cells define directionality of trunk, but not cranial, neural crest cell migration. Cell Rep. 15, 2076–2088. 10.1016/j.celrep.2016.04.067, PMID: 27210753 PMC4893160

[ref106] Riddiford N. Schlosser G. (2016). Dissecting the pre-placodal transcriptome to reveal presumptive direct targets of Six1 and Eya1 in cranial placodes. eLife 5:e17666. 10.7554/eLife.17666, PMID: 27576864 PMC5035141

[ref107] Rocha M. Singh N. Ahsan K. Beiriger A. Prince V. E. (2020). Neural crest development: insights from the zebrafish. Dev. Dyn. 249, 88–111. 10.1002/dvdy.122, PMID: 31591788 PMC7273345

[ref108] Roellig D. Tan-Cabugao J. Esaian S. Bronner M. E. (2017). Dynamic transcriptional signature and cell fate analysis reveals plasticity of individual neural plate border cells. eLife 6:e21620. 10.7554/eLife.21620, PMID: 28355135 PMC5371430

[ref164] Rogers C. D. Nie S. (2019). Specifying neural crest cells: from chromatin to morphogens and factors in between. Wiley Interdiscip. Rev. Dev. Biol. 7:e322. 10.1002/wdev.322, PMID: 29722151 PMC6215528

[ref109] Rothstein M. Simoes-Costa M. (2020). Heterodimerization of Tfap2 pioneer factors drives epigenomic remodeling during neural crest specification. Genome Res. 30, 35–48. 10.1101/gr.249680.119, PMID: 31848212 PMC6961570

[ref110] Sasai N. Mizuseki K. Sasai Y. (2001). Requirement of Foxd3-class signaling for neural crest determination in *Xenopus*. Development 128, 2525–2536. PMID: 11493569 10.1242/dev.128.13.2525

[ref111] Sato S. Ikeda K. Shioi G. Ochi H. Ogino H. Yajima H.. (2010). Conserved expression of mouse Six1 in the pre-placodal region (PPR) and identification of an enhancer for the rostral PPR. Dev. Biol. 344, 158–171. 10.1016/j.ydbio.2010.04.029, PMID: 20471971

[ref112] Sato T. Sasai N. Sasai Y. (2005). Neural crest determination by co-activation of Pax3 and Zic1 genes in *Xenopus* ectoderm. Development 132, 2355–2363. 10.1242/dev.01823, PMID: 15843410

[ref113] Sauka-Spengler T. Meulemans D. Jones M. Bronner-Fraser M. (2007). Ancient evolutionary origin of the neural crest gene regulatory network. Dev. Cell 13, 405–420. 10.1016/j.devcel.2007.08.005, PMID: 17765683

[ref114] Scerbo P. Monsoro-Burq A. H. (2020). The vertebrate-specific VENTX/NANOG gene empowers neural crest with ectomesenchyme potential. Sci. Adv. 6:eaaz1469. 10.1126/sciadv.aaz1469, PMID: 32494672 PMC7190326

[ref115] Schille C. Schambony A. (2017). Signaling pathways and tissue interactions in neural plate border formation. Neurogenesis 4:e1292783. 10.1080/23262133.2017.1292783, PMID: 28352644 PMC5358704

[ref116] Schlosser G. (2008). Do vertebrate neural crest and cranial placodes have a common evolutionary origin? BioEssays 30, 659–672. 10.1002/bies.20775, PMID: 18536035

[ref117] Schlosser G. (2010). “Making senses development of vertebrate cranial placodes” in International review of cell and molecular biology. ed. Jeon K. (Elsevier), 129–234. 10.1016/S1937-6448(10)83004-7 20801420

[ref118] Schlosser G. (2014). Early embryonic specification of vertebrate cranial placodes. Wiley Interdiscip. Rev. Dev. Biol. 3, 349–363. 10.1002/wdev.142, PMID: 25124756

[ref119] Schlosser G. Awtry T. Brugmann S. A. Jensen E. D. Neilson K. Ruan G.. (2008). Eya1 and Six1 promote neurogenesis in the cranial placodes in a SoxB1-dependent fashion. Dev. Biol. 320, 199–214. 10.1016/j.ydbio.2008.05.523, PMID: 18571637 PMC2671077

[ref120] Shellard A. Mayor R. (2019). Integrating chemical and mechanical signals in neural crest cell migration. Curr. Opin. Genet. Dev. 57, 16–24. 10.1016/j.gde.2019.06.004, PMID: 31306988 PMC6838680

[ref121] Sheng G. Stern C. D. (1999). Gata2 and Gata3: novel markers for early embryonic polarity and for non-neural ectoderm in the chick embryo. Mech. Dev. 87, 213–216. 10.1016/S0925-4773(99)00150-1, PMID: 10495290

[ref122] Simoes-Costa M. Bronner M. E. (2015). Establishing neural crest identity: a gene regulatory recipe. Development 142, 242–257. 10.1242/dev.105445, PMID: 25564621 PMC4302844

[ref123] Simoes-Costa M. Bronner M. E. (2016). Reprogramming of avian neural crest axial identity and cell fate. Science 352, 1570–1573. 10.1126/science.aaf2729, PMID: 27339986 PMC5100669

[ref124] Simões-Costa M. S. McKeown S. J. Tan-Cabugao J. Sauka-Spengler T. Bronner M. E. (2012). Dynamic and differential regulation of stem cell factor Foxd3 in the neural crest is encrypted in the genome. PLoS Genet. 8:e1003142. 10.1371/journal.pgen.1003142, PMID: 23284303 PMC3527204

[ref125] Simões-Costa M. Tan-Cabugao J. Antoshechkin I. Sauka-Spengler T. Bronner M. E. (2014). Transcriptome analysis reveals novel players in the cranial neural crest gene regulatory network. Genome Res. 24, 281–290. 10.1101/gr.161182.113, PMID: 24389048 PMC3912418

[ref126] Singh S. Groves A. K. (2016). The molecular basis of craniofacial placode development. Wiley Interdiscip. Rev. Dev. Biol. 5, 363–376. 10.1002/wdev.226, PMID: 26952139 PMC4833591

[ref127] Sittewelle M. Monsoro-Burq A. H. (2018). AKT signaling displays multifaceted functions in neural crest development. Dev. Biol. 444, S144–S155. 10.1016/j.ydbio.2018.05.023, PMID: 29859890

[ref128] Soldatov R. Kaucka M. Kastriti M. E. Petersen J. Chontorotzea T. Englmaier L.. (2019). Spatiotemporal structure of cell fate decisions in murine neural crest. Science 364:eaas9536. 10.1126/science.aas9536, PMID: 31171666

[ref129] Steventon B. Araya C. Linker C. Kuriyama S. Mayor R. (2009). Differential requirements of BMP and WNT signalling during gastrulation and neurulation define two steps in neural crest induction. Development 136, 771–779. 10.1242/dev.029017, PMID: 19176585 PMC2685944

[ref130] Steventon B. Mayor R. (2012). Early neural crest induction requires an initial inhibition of WNT signals. Dev. Biol. 365, 196–207. 10.1016/j.ydbio.2012.02.029, PMID: 22394485 PMC3657187

[ref131] Streit A. (2002). Extensive cell movements accompany formation of the otic placode. Dev. Biol. 249, 237–254. 10.1006/dbio.2002.0739, PMID: 12221004

[ref132] Streit A. (2018). Specification of sensory placode progenitors: signals and transcription factor networks. Int. J. Dev. Biol. 62, 191–201. 10.1387/ijdb.170298as, PMID: 29616729

[ref133] Streit A. Berliner A. J. Papanayotou C. Slrulnik A. Stern C. D. (2000). Initiation of neural induction by FGF signalling before gastrulation. Nature 406, 74–78. 10.1038/35017617, PMID: 10894544

[ref134] Streit A. Stern C. D. (1999). Establishment and maintenance of the border of the neural plate in the chick: involvement of FGF and BMP activity. Mech. Dev. 82, 51–66. 10.1016/S0925-4773(99)00013-1, PMID: 10354471

[ref135] Stuhlmiller T. J. García-Castro M. I. (2012). Current perspectives of the signaling pathways directing neural crest induction. Cell. Mol. Life Sci. 69, 3715–3737. 10.1007/s00018-012-0991-8, PMID: 22547091 PMC3478512

[ref136] Suzuki A. Ueno N. Hemmati-Brivanlou A. (1997). *Xenopus* msx1 mediates epidermal induction and neural inhibition by BMP4. Development 124, 3037–3044. PMID: 9272945 10.1242/dev.124.16.3037

[ref137] Sykes T. G. Rodaway A. R. F. Walmsley M. E. Patient R. K. (1998). Suppression of GATA factor activity causes axis duplication in *Xenopus*. Development 125, 4595–4605. PMID: 9806909 10.1242/dev.125.23.4595

[ref138] Szabó A. Mayor R. (2018). Mechanisms of neural crest migration. Annu. Rev. Genet. 52, 43–63. 10.1146/annurev-genet-120417-031559, PMID: 30476447

[ref139] Tambalo M. Mitter R. Wilkinson D. G. (2020). A single cell transcriptome atlas of the developing zebrafish hindbrain. Development 147:dev184143. 10.1242/dev.184143, PMID: 32094115 PMC7097387

[ref140] Teng L. Mundel N. A. Frist A. Y. Wang Q. Labosky P. A. (2008). Requirement for Foxd3 in the maintenance of neural crest progenitors. Development 135, 1615–1624. 10.1242/dev.012179, PMID: 18367558 PMC2562748

[ref141] Theveneau E. Mayor R. (2012). Neural crest delamination and migration: from epithelium-to-mesenchyme transition to collective cell migration. Dev. Biol. 366, 34–54. 10.1016/j.ydbio.2011.12.041, PMID: 22261150

[ref142] Theveneau E. Steventon B. Scarpa E. Garcia S. Trepat X. Streit A.. (2013). Chase-and-run between adjacent cell populations promotes directional collective migration. Nat. Cell Biol. 15, 763–772. 10.1038/ncb2772, PMID: 23770678 PMC4910871

[ref143] Thiery A. Buzzi A. L. Streit A. (2020). “Cell fate decisions during the development of the peripheral nervous system in the vertebrate head” in Current topics in developmental biology. ed. Peter I. S. (Cambridge, MA, United States: Academic Press, Elsevier) 127–167. PMID: 10.1016/bs.ctdb.2020.04.002 32450959

[ref165] Trevers K. E. Prajapati R. S. Hintze M. Stower M. J. Strobl A. C. Tambalo M. (2018). Neural induction by the node and placode induction by head mesoderm share an initial state resembling neural plate border and ES cells. Proc. Natl. Acad. Sci. U. S. A. 115, 355–360. 10.1073/pnas.1719674115 29259119 PMC5777083

[ref144] Tribulo C. Aybar M. J. Nguyen V. H. Mullins M. C. Mayor R. (2003). Regulation of Msx genes by a Bmp gradient is essential for neural crest specification. Development 130, 6441–6452. 10.1242/dev.00878, PMID: 14627721

[ref145] Vadasz S. Marquez J. Tulloch M. Shylo N. A. García-Castro M. I. (2013). Pax7 is regulated by cMyb during early neural crest development through a novel enhancer. Development 140, 3691–3702. 10.1242/dev.088328, PMID: 23942518 PMC3742149

[ref146] Vega-Lopez G. A. Cerrizuela S. Tribulo C. Aybar M. J. (2018). Neurocristopathies: new insights 150 years after the neural crest discovery. Dev. Biol. 444, S110–S143. 10.1016/j.ydbio.2018.05.013, PMID: 29802835

[ref147] Villanueva S. Glavic A. Ruiz P. Mayor R. (2002). Posteriorization by FGF, WNT, and retinoic acid is required for neural crest induction. Dev. Biol. 241, 289–301. 10.1006/dbio.2001.0485, PMID: 11784112

[ref148] Wilson P. A. Hemmati-Brivanlou A. (1995). Induction of epidermis and inhibition of neural fate by Bmp-4. Nature 376, 331–333. 10.1038/376331a0, PMID: 7630398

[ref149] Wilson S. I. Rydström A. Trimborn T. Willert K. Musse R. Jessell T. M.. (2001). The status of WNT signalling regulates neural and epidermal fates in the chick embryo. Nature 411, 325–330. 10.1038/35077115, PMID: 11357137

[ref150] Wynn M. L. Rupp P. Trainor P. A. Schnell S. Kulesa P. M. (2013). Follow-the-leader cell migration requires biased cell-cell contact and local microenvironmental signals. Phys. Biol. 10:035003. 10.1088/1478-3975/10/3/035003, PMID: 23735560 PMC3756809

[ref151] Xu H. Dude C. M. Baker C. V. H. (2008). Fine-grained fate maps for the ophthalmic and maxillomandibular trigeminal placodes in the chick embryo. Dev. Biol. 317, 174–186. 10.1016/j.ydbio.2008.02.012, PMID: 18367162

[ref152] Yardley N. García-Castro M. I. (2012). FGF signaling transforms non-neural ectoderm into neural crest. Dev. Biol. 372, 166–177. 10.1016/j.ydbio.2012.09.006, PMID: 23000357 PMC3541687

[ref153] Zhang J. M. Hoffmann R. Sieber-Blum M. (1997). Mitogenic and anti-proliferative signals for neural crest cells and the neurogenic action of TGF-β1. Dev. Dyn. 208, 375–386. 10.1002/(SICI)1097-0177(199703)208:3<375::AID-AJA8>3.0.CO;2-F, PMID: 9056641

